# The Neuro–Bone Axis in Metastatic Progression: Innervation, Neuro-Immune–Osteoclast Crosstalk, and Therapeutic Opportunities

**DOI:** 10.3390/biology15040364

**Published:** 2026-02-21

**Authors:** Mohamad Bakir, Alhomam Dabaliz, Mohammed Raddaoui, Hala Fatash, Nourhan Elsaadany, Wael AlKattan, Khalid Said Mohammad

**Affiliations:** 1 Department of Medicine, College of Medicine, Alfaisal University, Riyadh 11533, Saudi Arabia; mbakir@alfaisal.edu (M.B.); mraddaoui@alfaisal.edu (M.R.); hfatash@alfaisal.edu (H.F.); nelsaadany@alfaisal.edu (N.E.); 2 Department of Clinical Skills, College of Medicine, Alfaisal University, Riyadh 11533, Saudi Arabia; almdabaliz@alfaisal.edu; 3 Department of Surgery, College of Medicine, Alfaisal University, Riyadh 11533, Saudi Arabia; walkattan@alfaisal.edu; 4 Department of Anatomy, College of Medicine, Alfaisal University, Riyadh 11533, Saudi Arabia

**Keywords:** neuro–bone axis, bone metastases, sympathetic nerves, tumor–nerve crosstalk, β-adrenergic signaling, neuropeptides

## Abstract

Bone metastases represent a major cause of morbidity in advanced cancers, yet the neural regulation of metastatic growth within bone remains largely unexplored. The skeletal system is richly innervated by sensory and sympathetic nerve fibers that influence bone remodeling, hematopoiesis, and immune surveillance. Emerging evidence suggests that disseminated tumor cells exploit these neural circuits to create a growth-permissive microenvironment. Tumor-secreted neurotrophic factors can induce nerve sprouting, while sympathetic activation via β-adrenergic receptors promotes osteoclastogenesis, immunosuppression, and tumor proliferation. Neuropeptides such as substance P and calcitonin gene-related peptide exert dual effects on bone cells and infiltrating immune populations, further shaping the metastatic niche. The interplay between neural signals, osteolytic activity, and immune modulation positions the neuro–bone axis as a critical but underappreciated driver of metastatic progression. In this review, we synthesize current evidence on the anatomy and function of bone innervation, tumor-induced neural remodeling, and neuro–immune–osteoclast interactions. We highlight preclinical and clinical data supporting neuromodulatory strategies, including β-blockers, neurotrophin inhibitors, and targeted nerve ablation, as potential adjuncts to standard bone metastasis therapies. Finally, we identify key knowledge gaps, including the need for spatial and functional mapping of nerve–tumor interfaces and for integrating neuroimaging into bone metastasis detection. By framing the neuro–bone axis as a therapeutic target, we aim to catalyze interdisciplinary research that bridges oncology, neuroscience, and bone biology, with the goal of disrupting neural support for metastatic growth

## Simple Summary

Bone metastases are a frequent and debilitating complication of advanced cancers, particularly those of the breast, prostate, and lung, causing severe pain and fractures that drastically reduce a patient’s quality of life. While it is well understood that tumors invade the bone, the role of the body’s nervous system in facilitating this invasion has been largely overlooked. This review explores the “neuro–bone axis”, a network where nerves and tumor cells interact. Emerging evidence shows that tumors can manipulate nerves to grow into the cancer site and release stress signals that destroy bone tissue and suppress the immune system. The authors synthesize current research to demonstrate how this neural activity creates a fertile environment for cancer growth. The conclusion highlights that repurposing existing drugs, such as heart medications (beta-blockers) or pain relievers that block nerve signals, could interrupt this destructive cycle. By treating the nervous system as an active participant in cancer progression, new therapies can be developed to not only extend survival but also significantly reduce pain and skeletal complications for patients.

## 1. Introduction

Bone is the third most affected organ by metastasis. Breast, prostate, lung, renal, thyroid, and bladder cancers are the primary cancers most commonly resulting in skeletal involvement, which, in total, accounts for approximately 70% of metastatic bone disease (MBD) cases. When metastatic tumors move to the bone, they alter the skeletal framework, thus increasing the chances of skeletal-related events (SREs) that lead to serious complications for the patient’s health, such as bone pain of a severe nature, pathologic fractures, spinal cord compression, and hypercalcemia [1]. One of the most common signs of bone metastases is pain, which can be accompanied by neurological symptoms. All of these can cause significant deterioration in the quality of life and the level of the person’s functional capacity [2]. For instance, in lung cancer, about 30–40% of patients with metastatic non-small cell lung cancer (NSCLC) are reported to have bone metastases and, consequently, a high occurrence of skeletal-related events (SREs), thus highlighting the prevalence of skeletal complications in cancer cases [3]. Some of the serious manifestations, such as impaired mobility and bone marrow aplasia, also show how bone and bone marrow metastases can lead to systemic morbidity and result in a poor short-term prognosis [4]. Together, these complications result in significant patient suffering, increased use of the medical system, and shortened life expectancy in patients with late-stage cancers.

It is now well understood that many of these cancers that spread to the bone enter a state of dormancy before growing and developing into a clinically evident metastatic lesion [5]. This process was hypothesized as early as 1934 by Rupert Willis, an Australian pathologist, who suggested that dormancy was the key explanation to recurrent metastases observed in patients long after their remission from the primary disease [6]. These dormant tumor cells (DTCs) may remain in this state for several years before reactivation [5]. Thus, it is of utmost importance to explore the mechanisms behind this escape from dormancy to understand how to prevent it more effectively.

Among the mechanisms proposed is the effect of the nervous system, specifically the sensory and sympathetic nervous systems, on the niche. The effect of the nervous system on bones is described extensively in the literature, not only as an activator of osteolysis through stimulating the synthesis of receptor activator of nuclear factor κB ligand (RANKL) [7] but also as an inducer of angiogenesis through promoting the synthesis of vascular endothelial growth factor (VEGF) [8], as well as an interferer in the antitumor immune responses within the bone [8]. These effects have made the “neuro–bone axis” of great interest for exploration as a potential therapeutic target in the fight against bone metastasis, with many studies being conducted to investigate medications like β-blockers in the prevention and management of bone metastasis in cancers like breast cancer [9].

These effects have been described as separate inducers of DTC reawakening [7]. Therefore, exploring the role of the nervous system in the reactivation of DTCs can provide further insights that osteoclast-centric paradigms of dormancy escape may miss. This review provides a unifying, mechanistically grounded framework for understanding skeletal metastasis as a neuro–osteogenic–immune ecosystem rather than a tumor–bone interaction alone. Its novelty lies in integrating sensory and sympathetic neural circuits with osteoclast/osteoblast remodeling, vascular niche dynamics, and immune reprogramming to explain how neurogenic inflammation and adrenergic signaling can synchronize osteolysis, immune suppression, tumor persistence, and pain into self-reinforcing feed-forward loops. By organizing the field around actionable “intervention nodes” and highlighting cross-compartment therapeutic leverage points (tumor-intrinsic, nerve-directed, adrenergic, bone-targeted, immune-targeted, and vascular strategies), this review delivers a translational roadmap that clarifies where existing therapies may be repurposed, where combinations are biologically rational, and which mechanistic gaps should be prioritized to accelerate clinically effective, mechanism-based treatment strategies for patients with bone metastatic disease.

## 2. Anatomy and Physiology of Bone Innervation

### 2.1. Maps of Innervation

Bone has a highly organized nerve supply comprising sensory, sympathetic, and cholinergic nerve fibers, which are differentially distributed in the various skeletal compartments. Sensory peptidergic afferents, in particular those co-expressing calcitonin gene-related peptide (CGRP) and Substance P (SP), are most abundantly found in the periosteum, but they also reach the bone marrow and run through cortical channels such as Haversian canals, thereby forming a hierarchical pattern of sensory innervation [10,11,12,13]. These nerve fibers are often found running along blood vessels and spreading in areas that are changing or renewing; thus, they are considered to be involved in skeletal pain perception and act as a source of local signals [11,12]. Sympathetic adrenergic fibers delineated by tyrosine hydroxylase expression are also distributed across the periosteum, cortical bone, and marrow spaces. Here, they run along blood vessels and help regulate the autonomic control of the bone microenvironment [10,11,13].

**Figure 1:**
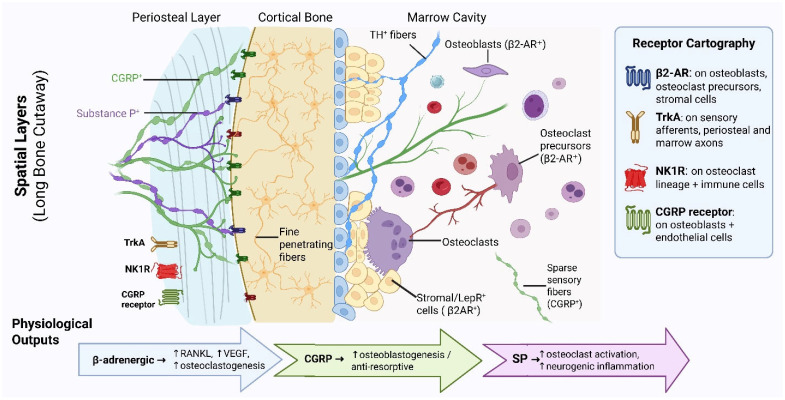
Anatomical and receptor map of the neuro–bone axis: This schematic cross-section of bone illustrates the spatial relationships among nerves, bone compartments, and key signaling receptors in the neuro–bone axis. It highlights the periosteum (outer bone layer), cortical bone, and marrow cavity, each innervated to varying degrees. Sensory nerve fibers (peptidergic nerves containing calcitonin gene-related peptide, CGRP, and substance P, SP) densely innervate the periosteum and extend into the marrow, while sympathetic nerve fibers (autonomic nerves) accompany blood vessels through the cortical bone into the marrow space. Key receptors mediating neuro–bone interactions are indicated: β2-adrenergic receptors (β2-AR) on osteoblasts and stromal cells respond to norepinephrine released by sympathetic nerves; TrkA (tropomyosin receptor kinase A), the high-affinity nerve growth factor receptor, is expressed on neural elements innervating bone; CGRP receptors on osteogenic and endothelial cells bind the sensory neuropeptide CGRP; and neurokinin-1 receptors (NK1R) on bone and immune cells bind the sensory neuropeptide SP. Neural signaling through these pathways regulates bone physiology, driving outcomes such as enhanced osteoclastogenesis, stimulation of angiogenesis, and initiation of neuroinflammatory responses. Created in BioRender. Mohammad, K. (2026) https://BioRender.com/cm2mnxq.

In addition to these well-known pathways, a recent study revealed cholinergic (VAChT^+^) axons in the periosteum, cortical bone, and marrow that arise from a postnatal neurotransmitter switch in a small population of sympathetic neurons [14]. Cholinergic signaling, classically associated with parasympathetic pathways, involves acetylcholine (ACh) acting through muscarinic receptors, while the sympathetic nervous system releases norepinephrine (NE), which binds to α- and β-adrenoreceptors on target bone cells. These cholinergic fibers interact structurally with osteocytes and osteolineage cells and influence bone formation and mechanosensitivity. There is further evidence of parasympathetic-like cholinergic signatures in bone and marrow, mediated through muscarinic receptor pathways. Nevertheless, the anatomical origin and identification of these fibers, as well as the presence of a genuine parasympathetic innervation of bone, remain a matter of controversy [15] (Figure 1).

### 2.2. Physiologic Roles of Bone Innervation

Bone remodeling, the process in adult bone that involves bone formation and resorption by osteoblast–osteoclast coupling, is tightly regulated and crucial for calcium metabolism and the repair of microfractures sustained during daily activity [16,17]. In recent years, multiple studies have focused on the neural regulation of bone remodeling and the interconnectedness of the skeletal and nervous systems.

The periosteum, the outer layer of the bone, along with other regions such as the bone marrow cavity, is highly innervated by peripheral nerves that primarily contain sensory and autonomic fibers. The autonomic nervous system (ANS) plays a crucial role in regulating bone remodeling by modulating osteoblast and osteoclast activity, thus maintaining skeletal homeostasis. This regulation is mediated by the release of numerous neurotransmitters, neuropeptides, and neural guidance factors, enabling communication between neurons regulated by the CNS and the cells of the bone microenvironment [17].

Paracrine stimuli such as CGRP, substance P, and neuropeptide Y can also be released by these skeletal nerve fibers, whereas hormones like leptin influence bone remodeling primarily through central and peripheral endocrine pathways rather than through direct release from nerve fibers [18].

The effect of hypothalamic-induced sympathetic activity on bone remodeling has been demonstrated in multiple studies, which show inhibition of bone formation and increased bone resorption [16]. “Clinical evidence indicates that the overstimulation of the SNS in patients with pheochromocytoma can significantly contribute to increased bone resorption, primarily affecting the trabecular bone through a weakened microarchitecture”, reports one study [17]. Another study linked bone density loss to diseases characterized by significant increases in SNS activity, such as reflex sympathetic dystrophy [19].

In the bone marrow, the SNS also innervates and regulates the hematopoietic stem cell (HSC) niche, playing a pivotal role in determining the fate, numbers, and function of HSCs. The SNS also governs the mobilization of HSCs. Norepinephrine released from sympathetic fibers controls Granulocyte colony-stimulating factor (G-CSF)-enforced HSC mobilization via regulation of the BM niche [20,21,22].

Furthermore, Nociceptive sensory neurons are required for enforced hematopoietic stem cell (HSC) mobilization, such as that induced by G-CSF. These neurons release CGRP, which acts directly on HSCs via CALCRL/RAMP1 to promote their release from the niche [20].

The decrease in bone formation is attributed to the release of the primary sympathetic neurotransmitter, norepinephrine, which is synthesized via the tyrosine hydroxylase pathway. Norepinephrine binds β2-adrenergic receptors expressed mainly on osteoblasts but also on osteoclasts [16,17,19]. This binding triggers the activation of cAMP/PKA, a signaling pathway that ultimately drives the upregulation of osteoblastic RANKL—the ligand that activates osteoclast differentiation and activity and suppression of osteoblastic OPG production [23]. Consequently, norepinephrine signaling would result in increased bone resorption and decreased bone formation, the release of calcium and phosphorus into the bloodstream, and decreased bone density [16,17,19].

Multiple studies have shown a link between β2-adrenergic receptor signaling by NE and cancer cell metastasis. In adult mice, stimulation of β2-adrenergic receptors in osteoblasts increased bone vascular density via β2AR-dependent induction of VEGF synthesis, thereby promoting breast cancer cell bone metastasis. This supports the potential for beta blockers as an adjuvant therapy to slow metastatic progression [9].

While norepinephrine is the primary sympathetic mediator of bone density, neuropeptide Y (NPY) acts as a critical co-transmitter, released from sympathetic fibers during periods of high physiological stress. NPY exerts a sophisticated, dual-compartment influence on skeletal homeostasis, with distinct mechanisms of action in the central nervous system (CNS) and the peripheral bone microenvironment. Peripherally, NPY’s role remains a subject of ongoing debate, largely due to receptor-dependent outcomes. Empirical evidence suggests that NPY can drive adipogenic differentiation of bone marrow-derived mesenchymal stem cells (MSCs) at the expense of the osteoblast lineage. This shift is mediated by inhibition of Y1R signaling, which downregulates key osteogenic transcription factors, including Tead1 and JunB, thereby increasing marrow adiposity and reducing osteoblast numbers. Conversely, some in vitro models demonstrate anabolic effects, where NPY binding to Y1R and Y2R receptors on osteoblasts stimulates proliferation. This suggests that the peripheral effects of NPY are highly context-dependent, potentially varying with local NPY concentrations and the specific receptor profiles expressed by bone cells across different developmental stages. In contrast to variable peripheral effects, the central action of NPY is widely recognized as a potent regulator of bone mass. Hypothalamic NPY signaling acts as a critical integrator of energy balance and skeletal health. High levels of hypothalamic NPY typically act via Y2 receptors to inhibit bone formation; consequently, the suppression or deletion of central NPY signaling has been shown to result in a high bone mass phenotype [17,19,23,24].

The main central regulation of bone mass is exerted through the leptin–hypothalamus–SNS–bone axis. Increased sympathetic signaling to the bone occurs when leptin, an adipocyte-derived hormone, binds its receptor in the arcuate nucleus of the hypothalamus. NPY neurons are then suppressed, and cocaine–amphetamine-regulated transcript neurons would be activated, thus increasing β2-adrenergic binding and bone resorption. In contrast, leptin’s peripheral effects on the bone would entail direct stimulation of osteoblastic differentiation and suppression of osteoclast formation, increasing bone density [19].

Coming to the effect of sensory fiber innervation, CGRP-positive nerves are the most distributed in the bone [25]. CGRP is stored in synaptic vesicles at the ends of sensory neurons and is released following depolarization [25,26]. Overall, CGRP has a positive effect on bone formation and healing processes. By signaling through the mitogen-activated protein kinase (MAPK) pathway and upregulating runt-related transcription factor (RunX2), CGRP promotes osteogenic differentiation. CGRP reduces apoptosis of osteoprogenitor cells and enhances their proliferation by inhibiting NF-κB activation, a signaling pathway that usually decreases osteoblast activity [11]. These mechanisms collectively enhance bone density and reduce bone resorption. Furthermore, CGRP plays a crucial role in bone fracture healing. Following an injury, it promotes bone angiogenesis and osteogenesis; thus, its failure to be released would impair fracture healing and delay fracture union [25].

Substance P is a tachykinin neuropeptide usually involved in pain perception and often co-released with CGRP in the bones. It enhances mesenchymal stem cell proliferation, promotes osteogenic differentiation, and activates signaling pathways involved in osteogenesis, such as MAPK. It also upregulates VEGF expression, promoting angiogenesis and bone formation. Substance P can directly regulate bone metabolism by binding the neurokinin-1 receptor (NK-1R), the high-affinity receptor for Substance P. A key feature of substance P is that it stimulates bone marrow stem cell proliferation in a dose-dependent manner, where low concentrations of substance P increase Runx2 expression and ALP activity (an early-stage osteogenic differentiation marker), while high concentrations promote matrix mineralization [11,24].

Sensory nerves, specifically those expressing CGRP, are enriched in the periosteum of metastatic bone. The crosstalk between tumor cells and sensory nerves, mediated by the CGRP receptor CLR/RAMP1, drives metastatic progression and is heavily implicated in cancer-induced bone pain. Elevated serum CGRP levels are associated with bone metastatic disease, highlighting the CGRP/CRLR axis as a potential therapeutic target [27].

## 3. Tumor-Induced Neural Remodeling (“Cancer Neurogenesis” in Bone)

### 3.1. NGF/TrkA Axis

The progression of tumor growth in bone is often associated with heightened pain intensity, primarily caused by the pathological remodeling of the peripheral nervous system. Investigations in prostate and breast cancer models reveal that TrkA-expressing sensory and sympathetic nerve fibers exhibit extensive sprouting and develop neuroma-like structures adjacent to cancer cells and altered bone. Ectopic calcitonin gene-related peptide (CGRP+), tropomyosin receptor kinase A (TrkA+), and growth-associated protein-43 (GAP43+) fibers proliferate due to the release of nerve growth factor (NGF) by tumor-associated stromal cells, which facilitates tumorigenesis and induces the reorganization of adjacent nerve structures [28,29].

Targeting this pathway through NGF sequestration or selective oral Trk inhibition (e.g., ARRY-470) has demonstrated efficacy in alleviating bone cancer pain and inhibiting ectopic sprouting. Although early continuous administration is typically the most effective for preventing pain development, late-stage intervention can still markedly diminish nociceptive behaviors and reverse established neuromas. The success in advanced stages has been attributed to the “resprouting” characteristic of tumor-associated nerves, enabling treatment to address the emergence of new cancer colonies [30,31].

In addition to pain, NGF signaling serves as a dual biomarker for pathological progression and neural integrity, governing cell survival and invasion in non-neuronal malignant tissues. This signaling serves as a crucial upstream mediator of bone repair and osteochondral differentiation post-trauma, facilitating essential axonal ingrowth. The systemic toxicity associated with NGF-targeted therapies underscores the necessity for localized intervention strategies to reconcile antitumor efficacy with the preservation of vital nerve innervation [32].

NGF and proNGF are best known for modulating neuronal activity via TrkA, p75NTR, and sortilin; however, their receptors are also widely expressed in non-neuronal malignant tissues. In breast and prostate cancers, these growth factors promote cell survival, proliferation, and invasion, with the TrkA signaling profile reflecting oncogenic pathways, including the epidermal growth factor receptor pathway. The findings suggest that the neuronal and non-neuronal roles of the NGF family are interconnected, establishing these molecules as essential biomarkers and therapeutic targets for metastasis and cancer-related pain [33].

In an experimental ulnar stress fracture, NGF expression is significantly increased in periosteal stromal progenitors and macrophages, promoting the emergence of CGRP+TrkA+ sensory fibers prior to vascularization and ossification. Inhibition of TrkA catalytic activity markedly diminishes these sensory fibers, impedes revascularization, and postpones callus development, indicating that skeletal sensory nerves serve as a vital upstream mediator of bone healing [34].

### 3.2. Periosteal Sprouting and DRG Involvement

In a study employing a syngeneic multiple myeloma (MM) mouse model, researchers discovered that periosteal nerve sprouting of calcitonin gene-related peptide (CGRP+) and growth-associated protein 43 (GAP43+) fibers coincides with the initiation of nociception. The blockade of this sprouting provided temporary relief, and an increase in periosteal innervation was further supported by samples from multiple myeloma cases. This research’s primary finding is the infiltration of metastatic multiple myeloma into the dorsal root ganglia (DRG), a previously unrecognized characteristic of the disease. This infiltration induces substantial alterations in gene expression within the dorsal root ganglia innervating the affected bone, particularly influencing pathways associated with the cell cycle, immune response, and neuronal signaling. Histological evidence indicated that multiple myeloma cells in the dorsal root ganglia lead to a reduction in vascularization and direct neuronal damage, which likely contributes to the onset of late-stage myeloma-induced bone pain (MIBP) [35].

In an intratibial mouse xenograft model, human multiple myeloma (MM) cells and osteoclasts collaborate to create an acidic bone microenvironment that precipitates significant multiple myeloma bone pain (MMBP). This process is triggered by the activation of acid-sensing ion channel 3 (ASIC3)-responsive sensory neurons and the augmented sprouting of calcitonin gene-related peptide-positive (CGRP+) sensory nerves. Prevention of acidification through the blockade of the vacuolar proton pump (V-ATPase) or through the use of an acid-sensing ion channel 3 (ASIC3) antagonist diminished neuronal excitation and markedly alleviated symptoms of pain [36].

A translational study of human and canine samples revealed that osteosarcoma (OS) induces de novo nerve infiltration, particularly in the most proliferative areas rich in mesenchymal stromal cells (MSCs) within the tumor. Mesenchymal stromal cells and osteosarcoma cells promote axonal growth via the secretion of interleukin-6 (IL-6) and brain-derived neurotrophic factor (BDNF), a process markedly enhanced by the acidic tumor microenvironment. The innervation associated with the tumor establishes a reciprocal loop in which nerves promote additional proliferation and migration of osteosarcoma cells, indicating that disrupting this nerve–tumor axis may present new therapeutic possibilities [37].

In bone cancer pain (BCP) models, tumor infiltration leads to a significant upregulation of the orphan G protein-coupled receptor GPR160 in small-diameter C-fiber neurons of the dorsal root ganglia (DRG). This expression results from epigenetic remodeling, characterized by diminished H3K27me3 and elevated H3K27ac modifications, which facilitate the binding of the transcription activator Sp1 to the Gpr160 promoter. Targeted knockdown or gene knockout of GPR160 diminishes neuronal excitability and significantly alleviates mechanical allodynia, as well as heat and cold hyperalgesia induced by the tumor [38].

Substance P (SP) and invading macrophages together drive the progression of pain in mice with cancer-induced bone pain (CIBP) through a two-phase neuroimmune process. Substance P, released from dorsal root ganglion (DRG) neurons via exocytosis, initiates the condition in the early phase and recruits type I macrophages into the sciatic nerves and dorsal root ganglia. In order to attract more macrophages during the advanced phase, the dorsal root ganglia release chemokines, particularly C-C Motif Chemokine Ligand 2 (CCL2) and C-C Motif Chemokine Ligand 3 (CCL3). This cycle can be successfully stopped by blocking the P-neurokinin 1 receptor pathway [39].

Osteoclast enrichment and increased expression of netrin-1 and its receptor are observed in metastatic bone lesions in a rat model of bone cancer pain (BCP). In the dorsal root ganglia (DRG), a signaling cascade involving focal adhesion kinase (FAK) and Rac family small GTPase 1/Cell division cycle 42 (Rac1/Cdc42) is activated by this netrin-1 signaling, which, in turn, promotes the pathogenic rise in calcitonin gene-related peptide (CGRP)-positive nerve fibers. These phosphorylation levels were successfully decreased, nociceptive nerve innervation was diminished, and pain behaviors were markedly improved after intrathecal delivery of deleted in colorectal cancer (DCC)-siRNA [40].

Netrin-1, the first axon guidance molecule found in vertebrates, is a secreted cue that either attracts or repels axons through the deleted in colorectal cancer (DCC) and neogenin receptors or the deleted in colorectal cancer/uncoordinated-5 (DCC/Unc5) receptor complex. Although netrin-1 contributes to the adult CNS’s inability to regenerate, it is upregulated in Schwann cells after peripheral nerve transection and promotes migration, cell proliferation, and peripheral nerve regeneration. Therefore, a new treatment approach to improve functional recovery and peripheral nerve healing is to target the netrin-1 signaling system [41].

Using a dorsal root ganglia (DRG)–gastric cancer cell co-culture model, researchers observed reciprocal interactions between cancer cells and neurites, in which silencing netrin-1 markedly inhibited the migratory, invasive, and navigational capabilities of cancer cells along adjacent nerves. The findings, corroborated by in vivo sciatic nerve invasion models, demonstrate that netrin-1 and its receptor neogenin function synergistically to facilitate neural invasion, implying that the inhibition of netrin-1 activity may serve as a viable treatment option for gastric cancer [42].

Bone tumors, such as myeloma, induce periosteal nerve sprouting and dorsal root ganglion remodeling via cortical bone degradation, the release of neurotrophic and inflammatory mediators (NGF, IL-6, BDNF, acid/ASIC3 pathways), and, in certain instances, direct tumor infiltration of the dorsal root ganglion. Netrin-1 serves as a pivotal chemotropic signal that activates FAK–Rac1/Cdc42 signaling in sensory neurons through DCC (and possibly neogenin), facilitating periosteal sprouting and nociceptive innervation, thus hastening the onset of bone cancer pain.

### 3.3. Sensory Nociceptors as Disease Modifiers

The pathogenesis of cancer-associated bone pain (CABP) is primarily influenced by an acidic bone microenvironment, resulting from cancer cell metabolism and heightened osteoclast activity [43]. In Lewis lung cancer (LLC) mice, this acidity results in the sensitization and activation of calcitonin gene-related protein (CGRP+) sensory neurons [44].

Critical to this procedure are acid-sensing nociceptors, namely transient receptor potential vanilloid 1(TRPV1) and acid-sensing ion channel 3 (ASIC3). These proteins are abundantly expressed in neurons that innervate the marrow cavity, including both small-diameter myelinated (Aδ) and unmyelinated (C) fibers [43,45]. In experiments, capsaicin and other agonists raise mechanical sensitivity and fiber activity, but proton pump inhibitors and genetic deletion of TRPV1 considerably reduce pain [44,45]. This establishes TRPV1 as a key player in bone pain and an essential therapeutic target.

Research has identified multiple critical mechanisms that regulate TRPV1 function in the dorsal root ganglia (DRG). For example, increased interleukin-6 (IL-6) levels promote nociceptor sensitization via an IL-6/JAK/PI3K/TRPV1 signaling pathway [46]. In contrast, programmed death-ligand 1 (PD-L1) functions as an intrinsic inhibitor by activating SHP-1, which diminishes TRPV1 currents and alleviates bone cancer pain [47]. Disrupting these signaling pathways or inhibiting the formation of the acidic environment provides a mechanism-based approach to managing CIBP [43,46].

In addition to pain perception, TRPV1 is essential for tumor–nerve interaction and cancer advancement [48]. In triple-negative breast cancer (TNBC), TRPV1-mediated signaling facilitates sensory neuron outgrowth; significantly, TRPV1 knockout in these animals not only postpones tumor progression but also diminishes lung metastasis [49]. In breast cancer models implanted into the tibia, activated sensory neurons secrete hepatocyte growth factor (HGF) through a TRPV1-dependent pathway, promoting further cancer colonization and dissemination [50].

The function of TRPV1 is sophisticated and encompasses immunological and skeletal wellness. It is expressed in multiple immune cells, including lymphocytes and macrophages, where its activation can initiate neurogenic inflammation [51]. Moreover, in periodontal tissue, the TRPV1-CGRP axis is crucial for averting bone loss by suppressing osteoclastogenesis [52]. Given that TRPV1 modifiers can affect cell proliferation, apoptosis, and the tumor microenvironment via both dependent and independent mechanisms, additional research is essential for optimizing these channels as pharmacological targets [53].

### 3.4. Candidate Guidance Cues

Human osteoblasts and osteoclasts consistently express netrin-1 and class 3 semaphorin mRNAs, indicating a local axon-guidance environment in bone that can attract sensory and sympathetic fibers [54]. In a rat model of bone cancer pain, metastatic tibial lesions exhibit elevated levels of netrin-1 and DCC, accompanied by the proliferation of CGRP+ nociceptive fibers; the knockdown of DCC diminishes neurite density and pain, suggesting that netrin-1–DCC–FAK/Rac1/Cdc42 signaling facilitates sensory neurite outgrowth within the bone tumor microenvironment [40]. Findings in pancreatic cancer indicate that netrin-1/neogenin signaling promotes sympathetic innervation, epithelial–mesenchymal transition (EMT), and metastasis, whereas inhibiting this pathway reduces nerve density and tumor burden [55]. Netrin-1 is extensively increased in malignancies, facilitating epithelial–mesenchymal transition, stemness, angiogenesis, and immune suppression; its inhibition (NP137) diminishes EMT, tumor proliferation, and therapeutic resistance in many models and preliminary clinical studies [56,57,58,59,60].

Semaphorin 4D (Sema4D), produced from osteoclasts, and its receptor, Plexin B1, impede osteoblast differentiation and are associated with bone metastasis; Sema4D is overexpressed in breast cancer cells and facilitates skeletal metastases, partly through osteoclast activation and osteoblast suppression [61,62]. In head and neck cancer, Sema4D expression is correlated with bone invasion and myeloid-derived suppressor cell recruitment, which connects immunosuppression, semaphorin signaling, and bone remodeling [63]. Sema4F can cause axonogenesis and perineural invasion in prostate cancer, directly promoting tumor neurogenesis, while class 3 semaphorins often prevent axon development in malignancies [63,64].

Beyond nociceptive sprouting, it is still mostly unknown as to whether netrin-1 or specific semaphorins directly route autonomic and sensory axons into bone metastases within in vivo bone-specific cancer models [13,34,35,37]. It is unclear how much DCC, UNC5, neogenin (netrin-1), and other plexin/neuropilin complexes (semaphorins) contribute to axon guidance, the remodeling of bones, and tumor cell actions in the bone niche [56,63,64,65,66].

Current research indicates that semaphorins, particularly Sema4D/F, modulate bone remodeling, innervation, and metastasis, and that netrin-1 is a pro-tumor and pro-sprouting cue in bone cancer pain models. The specific effect of this innervation on the development of bone cancer and direct proof that these chemicals serve as legitimate guidance cues for cancer neurogenesis within bone are, however, still significant and mostly unanswered problems.

### 3.5. Bidirectional Signaling

Current research demonstrates the crucial function of nerve–cancer interaction in promoting tumor development and spread among various solid tumor types [67,68]. Elevated intratumoral nerve density is associated with an unfavorable prognosis, as nerves penetrate the tumor microenvironment (TME) to promote cancer cell proliferation and dissemination [67,68,69]. This reciprocal interaction is facilitated by the secretion of neurotrophic growth factors, including nerve growth factor (NGF) and brain-derived neurotrophic factor (BDNF), released by cancer cells and carcinoma-associated fibroblasts (CAFs) to enhance axonogenesis and de novo innervation. In contrast, invading neurons secrete neurotransmitters, including noradrenaline and acetylcholine, which stimulate membrane receptors to promote angiogenesis and the proliferation of cancer stem cells [67,69,70].

This communication not only directly stimulates tumor cells but also influences the tumor stroma, where mesenchymal stromal cells (MSC) and extracellular acidosis enhance the release of pro-neurotrophic mediators such as interleukin-6 [67,70]. Moreover, the emerging idea of nerve dependency and perineural invasion, where cancer cells infiltrate adjacent nerves, offers an alternate pathway for metastasis and tumor-associated pain [68,70]. These essential discoveries collectively highlight the significance of the nerve–tumor axis and may establish a basis for creating effective anti-neurogenic medicines to impede tumor-induced neurogenesis [37,67,68,69].

Extracellular vesicles (EVs) generated from tumors contain proteins and miRNAs that reprogram neurons and stromal cells, promoting neurogenesis in premetastatic and metastatic bone niches [71,72]. These stimuli increase intratumoral and peritumoral innervation in bone by acting on sympathetic fibers, Schwann cells, neural progenitors, and periosteal sensory nerves [26,73,74].

Norepinephrine (NE) activates β2/β3 adrenergic receptors on tumors and osteoblasts, facilitating epithelial–mesenchymal transition (EMT), proliferation, migration, and the reactivation of dormant cells; in osteoblasts, β2 adrenergic receptor–HIF 1α–CXCL12 signaling amplifies prostate cancer invasion and EMT in bone [67,74,75]. Through muscarinic receptors (M1/M3/M4), parasympathetic acetylcholine (ACh) promotes tumor growth, metastasis, and neuroendocrine differentiation; cholinergic signaling can also activate YAP and the Wnt/β-catenin pathway, which expands cancer stem-like cells [75,76]. CGRP+ sensory neurons are more abundant in bone metastases; CGRP activates p38/HSP27 signaling in tumor cells by binding to CRLR, and therapy increases bone metastatic burden and proliferation [26,73]. The conventional “vicious cycle” of bone metastasis is further fueled by growth factors (TGF-β, IGFs) released by nerve-driven β-adrenergic signaling in osteoblasts and osteoclasts, which indirectly improve osteoclastogenesis, bone resorption, vascular density, and extracellular matrix remodeling [72,74,77].

NGF/Trk inhibition, β blockage, denervation, and IL-6 or CGRP blockade all exhibit preclinical promise; nevertheless, the best timing, combinations, and systemic side effects for osteosarcoma and bone metastases remain unclear [73,75,78,79].

**Figure 2:**
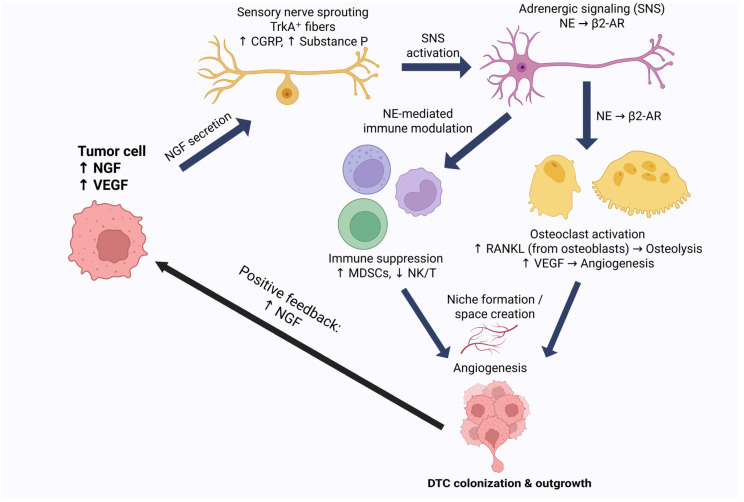
Tumor-driven remodeling of the neuro–bone axis: This schematic summarizes how invading tumor cells remodel the neuro–bone axis during bone metastatic progression, creating a feed-forward “vicious cycle” that supports cancer growth. Tumor cells in the bone microenvironment secrete nerve growth factor (NGF) and vascular endothelial growth factor (VEGF), which, together, promote robust sensory nerve sprouting (proliferation of new CGRP+ and SP+ nerve fibers) and increased angiogenesis (formation of new blood vessels). The expanding neural network and tumor presence also heighten sympathetic nerve activity, thereby increasing norepinephrine (NE) release. NE stimulation of β2-adrenergic receptors on osteoblast-lineage cells induces high levels of RANKL (Receptor Activator of NF-κB Ligand), which, in turn, drives osteoclast activation and bone resorption. Concurrently, these tumor-induced changes create an immunosuppressive niche in the bone marrow: myeloid-derived suppressor cells (MDSCs) are recruited, whereas anti-tumor immune effector cells, such as natural killer (NK) cells and T lymphocytes, are diminished in number or function. Collectively, the nerve outgrowth, bone breakdown, increased vasculature, and immune suppression facilitate the colonization and expansion of disseminated tumor cells (DTCs), leading to the establishment of a metastatic tumor niche in bone. A positive feedback loop ensues as the growing tumor (along with reactive stromal cells) continues to produce NGF (and other neurotrophic factors), further amplifying neural ingrowth and sympathetic signaling, which in turn sustains bone destruction and tumor progression. Created in BioRender. Mohammad, K. (2026) https://BioRender.com/7gj5uin.

Bidirectional interaction between nerves and tumors in bone is facilitated by neurotrophins, axon guidance cues, cytokines, extracellular vesicles, and acidity derived from tumors and stroma, which recruit and reprogram nerves. Additionally, neurotransmitters, neuropeptides, and chemokines released by nerves influence tumor, bone, and immune cells, thereby promoting growth, invasion, and metastasis. The basic structure of this positive feedback loop is evident; nonetheless, the specific wiring related to bone, the relative contributions of each pathway, and optimal therapeutic targeting remain significant unresolved challenges (Figure 2).

## 4. Neuro-Immune–Osteoclast Crosstalk in the Metastatic Niche

### 4.1. β-Adrenergic Signaling → Osteoclastogenesis and Angiogenesis

Neuroskeletal signaling reviews indicate that β-AR activation elevates RANKL expression in osteoblasts and osteocytes, hence promoting osteoclastogenesis and establishing RANKL-rich niches [22,79,80]. Activation of osteocytic β2-adrenergic receptors (clenbuterol, isoproterenol) elevates RANKL levels and the RANKL/OPG ratio, promoting osteoclastogenesis in cocultures [81,82]. Conditioned medium from activated osteoblasts stimulates angiogenesis ex vivo, and isoproterenol stimulation of β2-AR in osteoblasts increases Vegf-a expression and releases VEGF-A [83]. In vivo, β2-adrenergic receptor stimulation increases bone vessel density and VEGF-positive osteoblasts; genetic ablation of β2-adrenergic receptors in osteoblasts or inhibition of VEGF-A/VEGFR2 inhibits this neo-angiogenic response and diminishes breast cancer skeletal colonization [83,84]. A comprehensive mechanistic study indicates that β2-AR signaling in osteoblasts facilitates bone metastasis through a VEGF-dependent angiogenic switching and a RANKL-dependent enhancement of cancer cell homing and retention in bone [80]. Increased osteoblastic RANKL is a crucial mechanism linking adrenergic signaling to osteolytic lesions and metastatic development, as reviewed in sympathetic regulation of bone metastasis [85].

### 4.2. Neuropeptides

Substance P (SP)/Neurokinin-1 Receptor (NK1R) signaling constitutes a powerful pro-inflammatory and pro-cancer signaling pathway, whereas the RANKL/OPG system serves as the primary mediator of osteoclast-driven bone metastasis; nevertheless, direct mechanistic links have predominantly been demonstrated in inflammatory bone models rather than in cancer contexts.

Osteoclastogenesis and bone resorption are facilitated by elevated RANKL and/or decreased OPG, resulting in an increased RANKL/OPG ratio that activates RANK on osteoclast precursors through the TRAF6 → NF-κB, c-Fos, MAPK, NFATc1 signaling pathway [21,86,87,88].

Neoplastic cells in bone secrete PTHrP, TNF-α, IL-6, IL-11, and other cytokines that enhance RANKL expression and inhibit OPG in osteoblasts and stromal cells, establishing a “vicious cycle” of osteolysis and tumor proliferation [21,86,89,90,91]. SP/NK1R robustly stimulates MAPK (ERK, p38, JNK), NF-κB, and PI3K/Akt in neoplastic cells, promoting proliferation, inflammation, invasion, and survival [92,93,94]. Tumor cells and stromal/immune cells in metastatic bone express NK1R and produce pro-inflammatory cytokines in response to SP; these cytokines are known RANKL inducers in bone [95].

CGRP-mediated interactions from sensory neurons are believed to facilitate the development and advancement of metastatic niches in bone by directly activating tumor cells and altering immunological and bone-remodeling environments. CGRP+ sensory fibers densely innervate bones and calluses, regulating osteogenesis, angiogenesis, and immunological tone [96,97]. In osteosarcoma and bone metastasis, there is an elevation in periosteal/skeletal sensory innervation and CGRP signaling, which correlates with tumor proliferation, vascularization, and adverse prognoses [73,98,99]. CGRP derived from nociceptors exhibits significant immunomodulatory effects: it induces CD8^+^ T cell exhaustion in melanoma and influences an immunosuppressive milieu in medullary thyroid carcinoma through dendritic cell reprogramming [100,101]. Absence of CGRP in oral squamous carcinoma reduces tumor progression and correlates with increased infiltration by CD4^+^, CD8^+^, and NK cells, suggesting that CGRP typically constrains immunosurveillance [102]. In contrast, during acute injury, CGRP–RAMP1 signaling in neutrophils, monocytes, and macrophages facilitates the removal of inflammatory cells and enhances pro-repair macrophage polarization, hence expediting tissue and muscle recovery [103]. CGRP directly promotes tumor proliferation through the CRLR/RAMP1 pathway, activating p38/HSP27 and metabolic processes that facilitate the growth of metastatic seeds in bone and gastrointestinal tumors [104].

CGRP produced by sensory neurons serves as a primary organizer of metastatic niches by directly promoting tumor cell proliferation, modifying local immunity towards suppression or maladaptive repair, and regulating osteoblast–osteoclast dynamics and angiogenesis in bone. Targeting the CGRP receptor axis or its upstream sensory inputs is a novel approach to inhibit the formation of the bone metastatic niche and alleviate cancer-induced bone pain.

### 4.3. Neuro-Immune Modulation

Sympathetic fibers extensively innervate bone marrow; norepinephrine (NE) interacts with β-adrenergic receptors (β ARs) on hematopoietic stem cells (HSCs) and myeloid progenitors to facilitate myeloid proliferation and the generation of monocyte precursors that infiltrate malignancies as macrophages or myeloid-derived suppressor cells (MDSCs) [105,106,107].

When NE/β2 AR signaling activates STAT3 in myeloid cells, MDSCs are mobilized from bone marrow and differentiate into highly suppressive PMN, monocytic MDSCs, and tumor-associated macrophages. Additionally, VEGF, IL-4/IL-5, CCR4, and FoxP3-related pathways are upregulated [108]. Brief NE bursts exhibit anti-inflammatory properties, while persistent “subthreshold” stress increases Ly6C^hi inflammatory monocytes and fosters a tumor-promoting inflammatory environment [105]. The bias toward M2/repair phenotypes, along with the suppression of NFκB and pro-inflammatory cytokines, resulted in diminished antigen presentation and increased tumor proliferation [109]. The non-selective β-blocker propranolol inhibits β-adrenergic receptor–STAT3 activation in myeloid cells, therapy diminishing the generation of myeloid-derived suppressor cells and the differentiation of suppressive tumor-associated macrophages, partially reestablishing tumor immunity in various murine models and dogs [108,110].

In tumor-bearing mice, β-blockade has been demonstrated to enhance Th1-polarized, cytotoxic CD4 T cells, with the consequent anti-metastatic effect being specifically reliant on CD4 and monocytes [111]. Moreover, this blockade facilitates DC maturation, increases intratumoral effector CD8 T cells, and diminishes PD-L1^+ MDSCs/TAMs when used in conjunction with cancer vaccines [110]. In mouse models and retrospective patient cohorts, β blockade improves outcomes with checkpoint inhibitors, remodels the tumor microenvironment, and reverses catecholamine-driven systemic T cell dysfunction in intracranial tumors [112].

Data from mechanistic, preclinical, and early clinical investigations suggest that persistent SNS/NE signaling promotes bone marrow myelopoiesis toward MDSCs and immunosuppressive macrophages, while inhibiting effector T-cell activity. Pharmacologic β-adrenergic blockade can partially reconfigure this axis, reducing suppressive myeloid populations and augmenting CD4/CD8 T cell-mediated antitumor immunity, especially when used in conjunction with vaccines or checkpoint inhibitors. The specific roles of bone marrow–resident myeloid reprogramming relative to peripheral effects, as well as the optimal type, dosage, and timing of β-blockers, remain the focus of extensive investigation.

### 4.4. Conceptual Model

When bone and vascular niches shift from a high p38/low ERK to a permissive ERK-dominant state, produced by coupled osteolysis, angiogenesis, and immune suppression, disseminated tumor cells (DTCs) emerge from dormancy. Osteoclast-mediated resorption releases TGF β and other mitogens that promote tumor proliferation and perpetuate a “vicious cycle” of tumor development in bone [113].

Osteolytic breast and colorectal cancer metastases exhibit ERK1/2 activation in osteoclast precursors and osteoblasts; inhibiting MEK/ERK diminishes osteolysis and tumor proliferation [114,115]. This converts dormant DTCs from p38^high/ERK^low to ERK-driven proliferation [116]. Neoangiogenesis eliminates quiescence signals and provides VEGF and other growth factors that activate MAPK/ERK in perivascular disseminated tumor cells (DTCs), facilitating their awakening from dormancy in various organs [116,117,118].

In osteosarcoma lung colonization, host stromal growth factors enhance ERK phosphorylation and MCL1, facilitating early metastatic survival; ERK activity remains elevated in both early and established lesions [119]. Dormant DTCs are typically limited by interferon-driven STAT1 inhibitory signaling and active CD8 T cells. Bone marrow niches abundant in Tregs, MDSCs, and tolerogenic myeloid cells establish immunosuppressive settings that facilitate DTC survival and proliferation [116,117,120,121].

Myeloid-derived cytokines (IL-6, CXCL8, IL-4) concurrently facilitate osteoclastogenesis, angiogenesis, and MAPK/ERK activation in tumor and osteoclast precursors, thereby associating immunosuppression with ERK-mediated bone degradation and tumor proliferation [122,123,124].

Osteolytic remodeling, proangiogenic vascular alterations, and myeloid-driven immune suppression all depend on persistent MAPK/ERK signaling in tumor and stromal cells in both the lung and bone niches. DTC survival, emergence from dormancy, and metastatic outgrowth are enabled by this shift from a p38-dominated dormant state to an ERK-dominant milieu, underscoring ERK, its upstream cytokine networks, and the osteoclast–vasculature–immune axis as integrated therapeutic targets.

## 5. Evidence Linking Neural Activity to Bone Metastasis Burden

### 5.1. Preclinical Stress/SNS Activation Models

Isoproterenol and chronic stress facilitate skeletal colonization primarily by activating β_2_-adrenergic receptors (β_2_-AR) in osteoblast-lineage cells, thereby elevating VEGF and vascular/adhesion signals that enhance tumor cell seeding; non-selective β-blockers, such as propranolol, can significantly, though not entirely, mitigate these effects in preclinical models.

Norepinephrine/epinephrine and the β-agonist isoproterenol activate β_2_-AR on osteoblasts and bone marrow stromal cells (BMSCs), which are densely innervated by sympathetic fibers [80,85]. Stimulation of β_2_ adrenergic receptors in osteoblast-lineage cells enhances VEGF-A and other angiogenic factors, thereby augmenting bone vasculature and specialized “type H” capillaries that link angiogenesis and osteogenesis, establishing entrance points and supporting habitats for circulating breast cancer cells [80,85,125]. This stimulation also upregulates RANKL in osteoblasts/osteocytes, enhancing osteoclastogenesis and releasing growth factors from bone matrix that fuel metastatic growth [80,85,126]. Furthermore, it promotes IL-1β and endothelial E/P-selectin expression, improving tumor cell adhesion, extravasation, and retention in the bone marrow [85].

Chronic immobility and varying stress similarly stimulate sympathetic outflow; in mice, this enhances the efficacy of skeletal metastasis following tumor cell injection, independent of larger initial tumors, suggesting a microenvironmental influence [127]. β-blockers further safeguard bone density and specialized H arteries in scenarios of estrogen insufficiency or sympathoexcitation, facilitating long-term adjustment of the niche rather than immediate tumor eradication [125,126]. Epidemiological evidence about enhanced breast cancer outcomes associated with β-blocker use is inconclusive, suggesting that application to patients is hopeful yet not conclusive [128].

### 5.2. Sympathectomy/Denervation Studies

6-OHDA-induced sympathectomy exhibits both pro- and anti-metastatic effects, which are primarily influenced by the tumor model, organ site, and, particularly, the timing and extent of denervation, resulting in significant interpretive challenges for Pre-Metastatic Niche (PMN) investigations. In LLC/3LL lung carcinoma, the administration of systemic 6 OHDA, commencing two weeks prior to tumor implantation and persisting throughout the experiment, resulted in a reduction in sympathetic fibers, inhibition of tumor angiogenesis, decreased recruitment of lung myeloid-derived suppressor cells (MDSCs), and a reduction in lung metastasis, indicating that sympathetic nervous system activity facilitates the establishment of a pro-metastatic lung environment [129,130]. The administration of intraperitoneal 6 OHDA in an orthotopic breast cancer model, simulating nerve injury, expedited primary tumor growth and diminished tumor cell apoptosis. This nerve injury enhanced the proliferation and recruitment of tumor-associated macrophages (TAMs) and induced CD8^+^ T cell senescence through NFL–NF-κB signaling, facilitating immune evasion rather than immune protection [131]. In B16 F10 melanoma, administration of 6 OHDA prior to tumor injection eliminated peripheral sympathetic neural peak activity that closely correlated with metastatic progression; early denervation postponed metastasis, suggesting that intact sympathetic activity facilitates dissemination [132]. The timing of denervation in relation to tumor seeding (pre- versus post-implant) is a crucial determinant of metastatic outcomes. Pre-implant denervation can specifically inhibit PMN development, hence obstructing the creation of a conducive habitat for circulating tumor cells. Nerve injury in the presence of established tumors might unexpectedly promote growth by activating damage signals, such as neurofilament light chain (NFL), which may further boost the tumor microenvironment [133]. Tumor model and administration route (orthotopic versus subcutaneous; spontaneous versus experimental metastasis) determine the accuracy of modeling PMN creation and organotropism; numerous studies prioritize primary growth rather than authentic PMN biology [134,135].

### 5.3. Sensory Nerve Ablation

Bone-specific evidence suggests that sensory nerves and their neuromodulators typically facilitate the growth, vascularization, and dissemination of primary bone tumors, while targeted sensory denervation or neuromodulation can inhibit these processes in preclinical models. Significant deficiencies persist with respect to the subtype-specific, stage-specific, and systemic implications of these therapies. Specimens of human and canine osteosarcoma exhibit abundant intra- and peritumoral nerve fibers tightly linked with mesenchymal stroma and regions of substantial growth. Osteosarcoma-associated mesenchymal stromal cells and cancer-associated fibroblasts produce interleukin-6 and brain-derived neurotrophic factor, promoting axonogenesis; sensory innervation subsequently enhances osteosarcoma proliferation and migration, particularly in the acidic environment characteristic of aggressive osteosarcoma [37].

In TrkA knock-in mice, the functional suppression of TrkA+ sensory afferents decreased OS sensory innervation, VEGF-mediated vascularization, primary tumor proliferation, lung metastases, and extended survival; these effects were mirrored by bupivacaine liposomes and CGRP blocking (rimegepant) [98,136]. NGF facilitates the recruitment of M2 polarized macrophages and tumor proliferation; Trk inhibition (larotrectinib) counteracts these alterations in OS models [137]. In preclinical models, tumor-induced sensory neoneurogenesis and excitation promote the development of bone metastases and cause cancer-related bone pain; sensory denervation or inhibition decreased the burden of bone metastases and increased survival [138,139]. Nanoplatforms of local anesthetics (bupivacaine nanoparticles/liposomes) aimed to tumor or sarcoma innervation inhibit norepinephrine or CGRP signaling, diminishing macrophage infiltration and angiogenesis, and reducing tumor size, hence endorsing neuromodulation as a viable supplementary technique [98,136,140].

Most bone research emphasizes TrkA+/CGRP+ afferents; the relative roles of sympathetic/parasympathetic versus sensory fibers in bone malignancies and metastasis are inadequately understood [141]. Beyond osteosarcoma and selected examples of metastases, there is limited research on other primary bone tumors or solid tumors that invade bone; effects may vary based on tumor aggressiveness and the degree of inflammation [138,139]. In non-bone models, reviews point out that sensory denervation can occasionally accelerate metastasis through systemic neuro-immune alterations; comparable systemic trade-offs for bone-targeted denervation remain primarily unknown [141,142]. There is a notable lack of direct data regarding how the manipulation of bone nerves influences the initial seeding, dormancy, and subsequent reactivation of disseminated tumor cells in bone; current research on dormancy primarily emphasizes osteoblast–tumor interactions without specific consideration of neural regulation [143]. Although mechanistic connections (such as CGRP, NGF/TrkA, and IL-6/BDNF) are becoming more apparent, the optimal ways to combine them with immunotherapy, chemotherapy, or radiation therapy in bone contexts have not yet been determined [144].

## 6. Translational Therapeutics Targeting the Neuro–Bone Axis (Figure 3)

### 6.1. β-Blockers

Recent evidence on the role of β-adrenergic signaling in bone metastasis has prompted interest in the use of β-blockers to control it. One recent case report in 2017 found that propranolol was effective in maintaining control of a metastatic paraganglioma for 4 months after the discontinuation of the main therapy, which was temozolomide [145]. Additionally, in a study on dogs published in 2019, researchers found that single-agent propranolol or carvedilol inhibited cell viability and increased radiosensitivity in primary bone sarcoma cell lines [146]. The rationale behind this therapy most likely arises from the capacity of β-blockers to decrease HIF-inducible transcription targets, leading to decreased secretion of angiogenic factors such as VEGF, FGF, PDGF, EPO, and metalloproteases [147]. While the rationale may be strong, clinical evidence still seems to be mixed on the effect of β-blockers on cancer progression, as multiple meta-analyses have not been able to demonstrate a statistical significance in the cancer progression of patients on β-blockers [148,149,150], while other meta-analyses did demonstrate significant differences [151,152]. This underscores the need for additional prospective cohort studies with bone-specific endpoints to further validate and elucidate this potential treatment modality.

**Figure 3:**
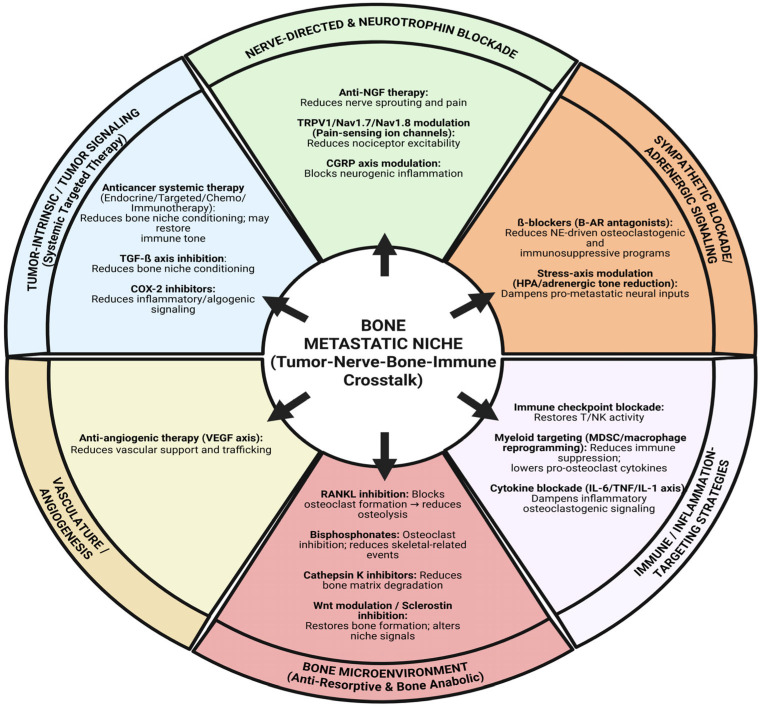
Therapeutic opportunities to disrupt tumor–nerve–bone–immune crosstalk in the bone metastatic niche. This schematic summarizes major intervention points within the bone metastatic microenvironment, organized by biological compartment and mechanism. The central hub represents the bone metastatic niche arising from reciprocal signaling among tumor cells, sensory/sympathetic nerves, bone-resident cells, immune populations, and the vascular niche. Surrounding wedges highlight therapeutic strategy classes: (i) tumor-intrinsic/tumor signaling approaches (systemic anticancer therapy and pathway-directed inhibition such as TGF-β axis and COX-2/PGE2-related inflammatory signaling); (ii) nerve-directed and neurotrophin blockade (anti-NGF therapy, modulation of nociceptor ion channels such as TRPV1/Nav1.7/Nav1.8, and CGRP-axis modulation) aimed at reducing nerve sprouting, excitability, and neurogenic inflammation; (iii) sympathetic/adrenergic signaling blockade (β-adrenergic antagonism and reduction in stress-axis adrenergic tone) to limit norepinephrine-driven osteoclastogenic and immunosuppressive programs; (iv) bone microenvironment targeting (RANKL inhibition, bisphosphonates, cathepsin K inhibition, and bone anabolic modulation such as Wnt/sclerostin pathway targeting) to suppress osteoclast activity, reduce osteolysis, and restore bone homeostasis; (v) immune/inflammation targeting (immune checkpoint blockade, myeloid-cell targeting or reprogramming, and cytokine blockade such as via IL-6/TNF/IL-1 pathways) to relieve immune suppression and dampen pro-osteoclast inflammatory cues; and (vi) vasculature/angiogenesis inhibition (anti-angiogenic strategies targeting the VEGF axis) to reduce vascular support and trafficking. Collectively, these options illustrate how multi-compartment targeting may interrupt feed-forward loops that sustain tumor growth, osteolysis, immune dysfunction, and pain in skeletal metastasis. Created in BioRender. Mohammad, K. (2026) https://BioRender.com/54n6x81.

### 6.2. NGF/TrkA Inhibition

The inhibition of NGF is not only explored as a possible disease-modifying therapy but also as a potential therapy for neuropathic pain, as NGF is an essential component implicated in its pathophysiology [153]. As discussed earlier, the NGF/TrkA signaling pathway has been investigated as an essential contributor to cancer development. As such, a preclinical trial targeting Trk family receptors with tyrosine kinase inhibitors such as Larotrectinib demonstrated strong synergistic effects with existing immunotherapies, achieving complete remission in melanoma [154]. However, it is important to note that clinical trials of anti-NGF antibodies were suspended by the FDA in the US due to adverse events, including rapidly progressive joint damage [155]. Phase 3 clinical trials were subsequently resumed and further confirmed the efficacy in relieving pain and improving joint function in patients with osteoarthritis [156,157]. Thus, while the efficacy of these drugs has been proven, their use must be undertaken cautiously as their adverse effects can be very severe. Peri-metastatic window testing may be necessary to assess the benefits of this treatment modality.

### 6.3. NK1R Antagonists (Substance P Pathway)

Recent studies have focused on the substance P pathway not only as a potential antitumor therapy but also as an anti-osteolytic therapy. Aprepitant, a NK-1R antagonist, has been shown to inhibit the viability of osteosarcoma cells in a dose-dependent manner in in vitro studies [158]. This study also found the ability of this drug to reduce the expression and activity of matrix metalloproteinases, angiogenic factors, and growth factors like NFκB, making it a possible inhibitor for osteolysis as well [158]. Combining aprepitant with chemotherapy has also shown to be protective against severe side-effects of chemotherapy against different organs as well as increase chemosensitization of osteosarcoma cells [159,160,161,162,163]. These antagonists have always been considered antiemetic drugs. Thus, repurposing it as an anti-cancer drug is pivotal to raising interest and awareness of this drug and promote more studies on its effects [164].

### 6.4. Interventional Neuromodulation

Thermal ablation of bone metastases, encompassing radiofrequency ablation (RFA), microwave ablation (MWA), and cryoablation, effectively alleviates pain by eradicating tumor tissue and peritumoral nociceptive fibers and receptors [165,166,167,168,169]. A prospective series of radiofrequency ablation (RFA) encompassing 206 patients demonstrates an instant enhancement in pain relief and quality of life (QoL) within three days, maintained for up to 12 months, with minimal device-related complications [170].

Bipolar radiofrequency ablation (RFA) for spinal metastases performed under local anesthetic results in a 75–80% reduction in visual analog scale (VAS) over 12 months, demonstrating high patient acceptability [166]. Moreover, systematic reviews of radiofrequency ablation (RFA) or microwave ablation (MWA) in conjunction with vertebroplasty, as well as spinal cryoablation, demonstrate substantial reductions in pain averaging 4–5 points on the visual analog scale (VAS) alongside effective local control and minimal major complication rates [167,171]. These are now conventional interventional oncology modalities in the multidisciplinary management of bone metastases, and are frequently used in conjunction with vertebroplasty, surgical interventions, or radiotherapy [172,173]. Guidelines and extensive studies on bone metastases emphasize radiotherapy, systemic therapy, surgery, and percutaneous bone/lesion ablation; surgical or chemical sympathectomy is not a standard, evidence-based approach for managing pain associated with bone metastases [173,174]. Interventional cancer pain studies highlight celiac, superior hypogastric, and sympathetic blocks for visceral abdominal or pelvic pain, rather than for skeletal metastases specifically [174]. Cordotomy, neuraxial medication delivery, and peripheral nerve blocks are options for refractory cancer pain; however, they are not specific to bone metastases and pose higher neurological risks [174]. 

Current radiotherapy guidelines clearly state that vertebroplasty, cryoablation, and radiofrequency ablation do not replace radiotherapy, highlighting their function as local adjuncts rather than independent neuromodulatory treatments [172].

### 6.5. Combinatorics

Persistent sympathetic signaling through β adrenergic receptors inhibits CD8^+^ T cells, enhances MDSC accumulation, and elevates Tregs, thereby fostering an immunosuppressive tumor microenvironment [175]. Non-selective β-blockers counteract this by decreasing MDSC levels and functionality, restoring CD8^+^ T cell effector activity, facilitating dendritic cell maturation, and augmenting responses to immune checkpoint inhibitors in preclinical models and clinical studies. Consequently, β-blockade pharmacologically alleviates stress within the systemic tumor-immune environment, promoting conditions favorable for T cell activity [175,176,177]. The CGA/CGRP sensory pathways are not extensively examined in MDSC biology; however, STING agonists exemplify the principle: neuro-immune modulation (of nociceptors) simultaneously enhances type I IFN signaling, diminishes bone degradation, and decreases local tumor load in bone cancer models, reinforcing the concept that targeting neural inputs can alter local immunity [178].

Bone metastases establish a “cold” microenvironment characterized by an abundance of MDSCs and regulatory T cells (Tregs), which diminishes the efficacy of immune checkpoint inhibitors (ICIs) [179,180,181]. Myeloid-derived suppressor cells (MDSCs) block T and natural killer (NK) cells through mechanisms involving arginase/iNOS, reactive oxygen species (ROS), transforming growth factor beta (TGF β), and direct inhibitory interactions [177,182,183,184,185,186].

Anti-resorptive drugs, like denosumab and zoledronate, elicit a complex therapeutic response by initially inhibiting RANKL–RANK-mediated osteoclast activation and osteolysis. This inhibition is essential as it prevents the production of TGF-β and other bone-derived immunosuppressive factors that generally impair the local immune response [179,180,181,187]. In addition to providing bone protection, these drugs also have a direct immunomodulatory effect; for example, the combination of denosumab with dual immune checkpoint inhibitors (ICIs) has been demonstrated to elevate IFN-γ and CXCL13 levels while redirecting peripheral T cells towards a CD8^+^ effector phenotype [179,187,188]. This simultaneous action results in clinical enhancements in overall survival (OS), progression-free survival (PFS), and response rates in individuals with NSCLC and melanoma with bone metastases, accomplishing these outcomes without considerable additional harm [179,180,181,187].

The objective is to combine neuromodulators (e.g., β-blockers) that inhibit adrenergic-induced MDSC proliferation with anti-resorptives that immunologically “re-warm” the bone marrow, thereby diminishing MDSC accumulation and reinstating T- and NK-cell effector functions, thereby enhancing the efficacy of ICIs and other immunotherapies in bone metastasis (Table 1).

## 7. Imaging and Measurement: How to See the Neuro–Bone Axis

### 7.1. Structural/Functional Imaging of Nerves

The presence of sympathetic innervation within the bone marrow, as demonstrated in murine studies [189,190], suggests a potential neural regulatory role in both hematopoiesis and bone physiology. This finding provides a rationale for developing and evaluating sympathetic-nerve-targeting tracers for bone and marrow imaging. Functional imaging of nerves enables analysis of the neuro–bone axis and directly detects sympathetic nerve activity within bone tissue. One such modality is [^11^C]HED PET, a technique traditionally used to assess neuroendocrine and cardiac disorders [191,192]. This approach uses a radiolabeled norepinephrine analog that binds to presynaptic sympathetic nerve terminals, thereby enabling visualization of sympathetic nerve activity and density. Notably, Franciuz et al. revealed that [^11^C]HED PET/CT can detect bone involvement in sympathetic nervous system tumors, highlighting its potential applications beyond conventional indications [193].

MIBG (^123^I) SPECT/CT is another established sympathetic-nerve imaging technique used in similar contexts. Combining a norepinephrine analog radiotracer with SPECT and CT allows functional and anatomical mapping of sympathetic nerve activity. Evidence has also demonstrated utility in identifying bone involvement in such neoplasms [194]. Collectively, these techniques can offer functional perspectives on nerve–bone interactions, not only in assessing tumor burden but also in clarifying the role of sympathetic innervation in tumor progression. Moreover, visualization of nerve–bone sympathetic activity enhances our understanding of these interactions.

Despite these advances, evidence remains limited regarding the ability of these imaging approaches to map sympathetic innervation in normal, non-diseased bone tissue. Tumor tracer uptake does not necessarily reflect physiological nerve distribution, and accurate interpretation is further challenged by several factors, including the low baseline nerve density within bone, limited spatial resolution, tracer kinetics, and technical constraints.

### 7.2. Tissue Clearing and Light-Sheet Microscopy

Mapping the structural architecture of nerves represents a major advancement in visualizing nerve–bone interactions. Advanced tissue-clearing techniques, such as various Dimensional Imaging of Solvent-Cleared Organs (DISCO)-based methods, combined with light-sheet microscopy, enable three-dimensional visualization of nerves in intact bones at cellular resolution. These approaches reveal the spatial relationships among bone cells, blood vessels, and bone marrow [195].

Emerging workflows combine traditional DISCO methods with light-sheet microscopy to enhance antibody labeling, tissue transparency, and imaging depth in dense bones such as the femur, knee, and bone marrow. For example, Thai et al. demonstrated that these methods can image sensory and sympathetic fibers in the femur and knee of mice [196]. By imaging sensory and sympathetic fibers in bone tissue, these workflows provide a framework for linking nerve architecture to bone disease. Moreover, advanced tissue-clearing methods provide quantitative analysis of nerve density and distribution throughout bone tissue. This can facilitate comparisons between healthy and diseased bones and assess the effects of different treatments.

### 7.3. Spatial Omics

Spatial omics is an innovative technique for mapping the spatial distribution of molecules within tissues [197]. One common approach involves mapping gene expression directly onto tissue sections using spatially barcoded RNA probes, allowing researchers to determine where specific genes are active [198]. This method can uncover cell–cell interactions, tissue organization, and microenvironmental dynamics. Moreover, spatial transcriptomics is particularly significant in understanding tumor progression [199] as it enables in situ profiling of tumor, stromal, and immune cells, revealing spatial heterogeneity [200].

In the context of bone tissue, it is especially useful for mapping interactions among tumor cells, osteoblasts, osteoclasts, bone marrow stromal cells, and sensory nerves, providing insights into tumor growth, bone metastasis, bone remodeling, and associated pain signaling. Spatial omics has demonstrated utility across multiple cancer types [201], with recent studies further highlighting its potential in musculoskeletal contexts [202,203,204,205].

However, evidence for its application in bone metastases remains limited. Currently, there are no comprehensive spatial atlases that map tumor cells, bone-resident cells, and infiltrating immune or nerve cells within metastatic bone lesions. Such maps would be crucial for understanding how tumor cells interact with key cellular and neural components of the bone microenvironment. They would additionally help identify spatially defined niches that drive tumor progression, bone remodeling, and pain signaling. Addressing this gap could not only uncover novel therapeutic targets and mechanisms of resistance but also facilitate the translation of spatial omics findings into clinical strategies for bone metastases, including mapping tumor–cell interactions, biomarker discovery, therapy response, and resistance.

### 7.4. Pain as a Proxy

Pain has traditionally been used as an indirect marker of nerve involvement in bone tumors, but it remains a poor indicator of actual nerve–bone activity. It is inherently subjective, making objective quantification impractical [206]. Moreover, its interpretation is often confounded by the effects of analgesic medications [207]. Although analgesics such as opioids may provide temporary symptomatic relief, they do not address the underlying malignancy. As a result, it may appear to reflect tumor regression, despite the absence of a true antitumor effect. Some individuals also adapt to chronic pain, resulting in underreporting despite ongoing or worsening pathology. Furthermore, pain offers no spatial information about where or how nerves interact with bone, and clinical symptoms often fail to correlate with structural, cellular, or molecular changes.

These limitations underscore the need for objective tools that directly assess nerve–bone interactions. Advanced imaging, spatial omics, and neural tracers offer precise measurements of nerve fiber spatial organization, density, and functional properties within bone tissue, revealing neural contributions to pathology that remain overlooked when relying on subjective pain assessments alone. Considering this, pain remains an important patient-centered outcome, but it should not serve as primary evidence of biological activity [208]. Instead, quantitative, mechanistic measurements should form the basis of trial endpoints, as reliance on pain alone can be misleading and may contribute to diagnostic or therapeutic errors.

## 8. Clinical Syndromes at the Neuro–Bone Interface

### 8.1. Cancer-Induced Bone Pain (CIBP)

Cancer-Induced Bone Pain (CIBP) refers to the pain caused by bone metastases or severe and common pain caused by primary bone tumors. This pain is well-documented to be the most important factor that reduced the quality of life in patients with metastasized cancer [28]. Many mechanisms have been shown to influence the development of this condition. Rodent models revealed pathological sprouting and neuroma formation by sensory and sympathetic nerve fibers in the periosteum and bone marrow of the metastatic tumor-invaded bone [209,210]. NGF was attributed as a major driver of this pathological sprouting as well as the neuroma formation; thus, studies on the use of anti-NGF were conducted, revealing markedly attenuated nerve sprouting as well as neuroma formation and ultimately bone pain [211,212].

### 8.2. Stress, Circadian SNS Tone, and Metastasis Risk

The effects of the SNS on the bone extends to changes in hematopoietic cell release and hematopoiesis [213]. It is well documented that administration of norepinephrine in murine models leads to elevation in both myeloid and lymphoid cell lines in the blood within minutes [214,215]. It has been found that norepinephrine is a major regulator of CXCL12 synthesis, a prime inhibitor of hematopoietic stem and progenitor cell (HSPC) migration and proliferation, it also induces retention of neutrophils [213]. This leads to a state of elevated HSPC cycling and expansion as well as elevated release of neutrophils within the circulation [213]. On the other hand, chronically elevated norepinephrine levels associated with chronic stress have been shown to negatively affect the immune system as well as enhance immunopathological responses [216]. This elevated level of inflammation is an important factor to consider in patients undergoing surgery for treatment of bone metastasis. Surgery induces a high-stress environment in the body, reducing the immune system’s activity and ability perioperatively. Thus, studies exploring different perioperative managements like anti-inflammation and blood transfusion should be done to help prevent vital organ injury and further metastasis during the surgery.

## 9. Knowledge Gaps and Prioritized Research Agenda

While the field of interactions between the nervous system and bone in bone metastasis has recently garnered significant attention, many facets remain to be studied. An important next step to understand the nervous system’s effects on cellular components within the bone is to compile and document, in an atlas, the β-adrenergic and neuropeptide receptors specific to different cell types within the bone. Another facet worth exploring is the change in the effect of neuromodulation on bone metastasis depending on the timing of its administration, whether in the peri-metastatic phase or in established lesions. This would help determine whether neuromodulation-based management should be considered a treatment or is better suited to prophylaxis. To fill these knowledge gaps, trials with bone-related endpoints must be conducted. These endpoints can include skeletal-related events, neuropeptide levels, and changes in imaging that focus on the neuro–bone axis, like the aforementioned [^11^C] HED PET/CT as well as MIBG (^123^I) SPECT/CT scans. Long-term studies on management related to these mechanisms will enable a better understanding of the safety profile and the oncology-specific risk–benefit of these treatment modalities.

## 10. Conclusions

The evidence presented supports that neural signaling within the bone microenvironment is not merely a byproduct of pain biology but constitutes a legitimate metastatic enabling system with numerous druggable targets. This neuro–bone axis promotes disease progression via an intricate feedback loop in which tumor-induced neural remodeling and neurotransmitter release directly affect skeletal colonization and the creation of permissive niches. The NGF-sprouting mechanism is central to this system. It is where nerve growth factor from tumors causes sensory and sympathetic fibers to reorganize in a way that causes both chronic pain and tumor growth. Moreover, sympathetic activity is a key factor in the “vicious cycle” of metastasis. The osteoblast β2-AR → VEGF/RANKL axis is what holds this together. Adrenergic stimulation of the osteoblast lineage expands the bone vasculature and increases osteoclastogenesis, thereby facilitating cancer cell trafficking to the bone and the release of growth factors from the bone matrix. In addition to these structural changes, neural inputs have a big effect on the local immune landscape. For example, persistent SNS/NE signaling creates an immunosuppressive environment characterized by abundant MDSCs and deficient T/NK cell effector function. Ultimately, these results indicate that important treatment options exist when transitioning from traditional palliative care to interventional neuro-oncology. By combining neuromodulators, such as beta-blockers or CGRP antagonists, with established anti-resorptives and immunotherapy, physicians might be able to “de-stress” the bone marrow with drugs and break the ERK-dominant state that allows dormant tumor cells to grow. Addressing the specific wiring of the bone–nerve–tumor axis is crucial for improving survival and quality of life in patients with metastasis.

## Figures and Tables

**Table 1:** Key neuro–bone axis mediators, pro-metastatic mechanisms, and therapeutic leverage points relevant to bone metastasis.

Pathway/Mediator	Primary Responding Cells/Receptor(s)	Key Effect(s) in Bone Metastasis	Representative Therapeutic Leverage Points	Evidence Level in Bone Metastasis
Sympathetic NE (SNS tone)	Osteoblast-lineage/stroma (beta2-AR); myeloid cells (beta-AR)	RANKL up; osteoclastogenesis/osteolysis; angiogenic support; immune suppression (MDSC skewing)	beta-blockers; stress/SNS dampening; circadian/SNS modulation	Strong preclinical; mixed observational human data; limited prospective testing
NGF/TrkA-driven sensory sprouting	Sensory afferents (TrkA); periosteal/marrow niche	Nerve sprouting; neurogenic inflammation; pain; can couple to vascular remodeling and niche permissiveness	Anti-NGF antibodies; TrkA/Trk pathway inhibition; local neuromodulation/ablation (selected lesions)	Robust CIBP biology; metastasis-burden data emerging, context-dependent
Substance P (SP) signaling	Osteoclast lineage, immune cells, tumor cells (NK1R)	Pro-osteoclastogenic/inflammatory signaling; supports motility and a permissive niche	NK1R antagonists (aprepitant-class) and rational combinations	Mechanistic and preclinical evidence; translational repurposing potential
CGRP pathway	Osteogenic/endothelial cells (CGRP receptor: CALCRL/RAMP1); sensory fibers	Dual biology: bone-active peptide with prominent nociception/neuroinflammation roles in CIBP	CGRP-blocking antibodies/gepants (pain-oriented); neuromodulation approaches	Limited direct metastasis evidence; stronger pain biology evidence
Osteolysis feed-forward loop	Osteoclast lineage (RANK); bone matrix factor release upon resorption	Osteolysis releases growth factors (e.g., TGF-beta) sustaining tumor outgrowth and remodeling programs	Denosumab; bisphosphonates; backbone therapy for combination with neuro-immune targeting	Strong clinical evidence for skeletal-related event reduction; disease-modifying effects vary

## Data Availability

No new data were created or analyzed in this study. Data sharing is not applicable to this article.
